# Prevalence of Sleep Disturbances in Latin American Populations and Its Association with Their Socioeconomic Status—A Systematic Review and a Meta-Analysis

**DOI:** 10.3390/jcm12247508

**Published:** 2023-12-05

**Authors:** F. A. Etindele Sosso, Filipa Torres Silva, Rita Queiroz Rodrigues, Margarida M. Carvalho, Sofia Zoukal, Gabriel Cordova Zarate

**Affiliations:** 1 Department of Global Health and Ecoepidemiology, Redavi Institute, Montréal, QC H4J 1C5, Canada; 2 Pneumonology Department, Centro Hospitalar de Trás-os-Montes e Alto Douro, 5000-508 Vila Real, Portugal; aftsilva@chtmad.min-saude.pt (F.T.S.); aqrodrigues@chtmad.min-saude.pt (R.Q.R.); margaridamc1305@gmail.com (M.M.C.); 3 Laboratory of Epidemiology, Faculty of Medicine and Pharmacy of Casablanca, University Hassan II, Casablanca 20250, Morocco; sofi.cap@gmail.com; 4 Faculté de Médecine de L’université Paris-Saclay, 75015 Paris, France; corzar91@gmail.com

**Keywords:** environment, South America, Central America, Latin America, mental health, socioeconomic status, sleep, public health, systematic review

## Abstract

Background: The worldwide increase in the prevalence and incidence of sleep disturbances represents a major public health issue. Among multiple determinants affecting sleep health, an individual’s socioeconomic status (SES) is the most ignored and underestimated throughout the literature. No systematic review on the relation between SES and sleep health has been previously conducted in Latin America. Methods: PRISMA guidelines were used. Results: Twenty articles were included in the final sample (all cross-sectional studies), and twelve among them were rated as fair or poor quality. Among these studies, 80.0% (n = 16) were performed in Brazil, 10.0% (n = 2) were performed in Peru, 5.0% (n = 1) were performed in Chile, and 5.0% (n = 1) were multicentric (11 countries). The combined total number of participants was N = 128.455, comprising 3.7% (n = 4693) children, 16.0% (n = 20,586) adolescents, and 80.3% (n = 103,176) adults. The results show the following: (1) The sleep outcomes analyzed were sleep duration, sleep quality/sleep disturbance, insomnia, excessive daytime sleepiness (EDS), obstructive sleep apnea (OSA)/sleep-disordered breathing (SDB) symptoms, and bruxism. (2) The most used determinants were income, education level, employment status/occupation, wealth/assets, and composite indices. (3) Higher SES was associated with shorter sleep duration. (4) Lower SES was associated with a decrease in sleep quality, less frequent snoring, more prevalent EDS, and sleep bruxism. (5) Lower education was associated with insomnia. (6) Higher education was associated with more sleep bruxism. (7) The pooled prevalence using a meta-analysis of the random effects model was 24.73% (95%CI, 19.98–30.19), with high heterogeneity (I^2^ = 100%). (8) The prevalence of sleep disturbances decreased with high education (OR, 0.83; 95%CI, [0.69–0.99]; I^2^ = 79%), while it increased with low income (OR, 1.26; 95%CI, [1.12–1.42]; I^2^ = 59%), unemployment (OR, 2.84; 95%CI, [2.14–3.76]; I^2^ = 0%), and being a housewife (OR, 1.72; 95%CI, [1.19–2.48]; I^2^ = 55%). Discussion: This meta-analysis shows that lower SES (education, income, and work) was associated with sleep disturbances in Latin America. Therefore, sleep disturbance management should be addressed with a multidimensional approach, and a significant investment in targeted public health programs to reduce sleep disparities and support research should be made by the government before the situation becomes uncontrollable.

## 1. Introduction

Promoting a healthy sleep is an ongoing project which never ends. Sleep management, with our modern lifestyle, is a complex public health outcome requiring multidimensional interventions at the economic level, the populational level, and the societal level [1,2]. Mental health is also highly dependent on sleep, which has a recognized impact on several brains functions [3] and global health status, as well as stress [4]. In addition, sleep has a significant influence on mental health due to its relationship with people’s socioeconomic status (SES) and its connection with multiple biological systems involved in neurological disorders, such as anxiety [1,5,6,7,8,9,10]. In others words, sleep disturbances are mental disorders resulting from complex socioecological and economic interactions between the brain, the society in which we live, global health, and SES [7,11,12]. Thus, sleep health inequalities represent a mental health outcome similar to the public health issues previously reported for cardiovascular, mental health, and metabolic diseases [11]. The empirical literature in Western countries seems to validate the hypothesis that low-SES individuals reported more sleep disturbances than high-SES people [13,14,15,16,17,18]. Similar evidence exists in Asia [19,20] and Oceania [21,22]. Thus, sleep health disparity could be a public health issue in other areas, like Africa and Latin America. Few studies have documented sleep health disparities in South and Central America due to a wide range of determinants, such as employment, income, education, occupation, and social position [11,12,23]. An exhaustive evaluation of the public health literature related to Latin America revealed that no systematic review on the relation between SES and sleep health has been previously conducted in Latin America. It is important to analyze if trends related to the influence of SES determinants of mental health on sleep health observed in Western countries are following the same patterns as in Latin America. The goals of this systematic review are to (1) document the global prevalence of sleep disturbances in Latin American populations and (2) document the influence of SES on different types of sleep disturbances.

## 2. Methods

### 2.1. Literature Search

**Figure 1:**
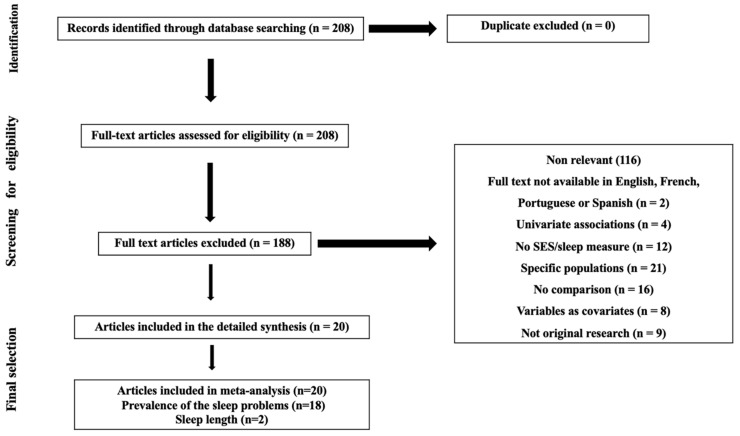
PRISMA flowchart of study selection process.

Relevant peer-reviewed studies included in this systematic review were identified by searching the databases Web of Science, Scopus, PubMed/Medline, and Google scholar between January 1990 and July 2022. A combination of the terms “socioeconomic”, “socio-economic”, “social position”, “social class”, “socioeconomic position”, “determinant*”, “indicators*”, “markers*”, “health inequalities”, “sleep”, “sleep disorders”, “sleep disturbances”, ‘’sleep complains”, “sleep outcome”, “sleep health”, “south america*”, “central america*”, and “latin america” was used. All included articles were identified following the PRISMA guidelines detailed in Figure 1 [24].

### 2.2. Inclusion and Exclusion Criteria

Empirical studies were defined as those of any design (cross-sectional, retrospective, or longitudinal) assessing humans of any gender, race/ethnicity, and age from the general population of any country from Latin America. The article should include an objective or a subjective measure of SES, such as income (monthly personal income, monthly family income, per capita income, and annual household income), educational level, wealth, profession/occupation, employment status, and perceived SES or self-reported SES. Proxy measures of SES, such as neighborhood and social class, were also included. Every sleep component, like sleep duration and sleep quality; and every sleep disturbance, like insomnia, excessive daytime sleepiness (EDS), obstructive sleep apnea (OSA), sleep-disordered breathing (SDB) symptoms, and bruxism, were considered dependent variables. For articles with samples composed of children and adolescents, perceived family SES measures, such as parental education, parental occupation, or annual household income, were used instead. Studies were excluded based on the following criteria: (1) they were interventional trials, every type of reviews (narrative, overview, systematic, umbrella, and meta-analyses), case series, case reports, conference series, or any writing without original research (editorial, commentary, letter to editors); (2) they were articles that did not provide statistical significance in cases where either SES or sleep was evaluated as a covariate or mediator; (3) the full text was not accessible; (4) the authors/researchers recruited participants with medical conditions at the beginning of study (for example, people with medications, including sleep pills; people with cancers; people with neurodegenerative diseases, etc.); and (5) articles that were not written in English, French, Portuguese, Arabic, or Spanish (the languages of the authors).

### 2.3. Selection of Evidence and Data Extraction

Two reviewers (FAES and SZ) independently reviewed the titles and abstracts of the studies identified by the search strategy and determined eligibility for inclusion, while disagreements were resolved by consensus with a third external reviewer. For studies that passed the initial screening, the entire text was collected, and the screening process was repeated by the same co-authors to conclude with the final articles included, validated by the third reviewer. Then, four reviewers (FTS, RQR, MC, and FAES) extracted from each report the following study characteristics: population, % women, age, sample size, SES measures, relevant statistics, interaction or mediation, sleep measures, conclusions/main effects, statistical methods, and results’ significance.

### 2.4. Quality Rating of Studies

The National Institute of Health’s (NIH) Quality Assessment Tool for Observational Cohort and Cross-Sectional Studies was used to rate the quality of the included studies [25]. This quality rating tool was used to analyze fourteen quality criteria, asking an equal number of questions about study objectives, population, exposures, outcomes, follow-up rates, and statistical analysis. SES was considered the exposure variable, and sleep measures the outcome variable, respectively. Overall quality ratings were calculated by taking the proportion of positive ratings on the sum of applicable criteria. Studies with a <50% positive rating were judged as poor quality, ≥65% as good quality, and the rest as fair quality [25]. 

### 2.5. Study Outcomes

The primary outcomes were (1) to determine the prevalence of sleep disturbances (sleep duration, sleep quality, insomnia, EDS, OSA/SDB symptoms, and bruxism) in Latin American populations and (2) to document the influence of SES (income, education, employment/occupation, wealth/assets, and composite indices) on different type of sleep disturbances. These were explored through an analysis of education, income, employment status, and perceived SES, if they were reported by at least two independent studies. The secondary outcomes were to (1) analyze the relationship of our findings with current public health literature and (2) promote a multidimensional approach for sleep management.

### 2.6. Data Analysis

A meta-analysis was performed using the meta package on R with RStudio interface (Version 4.1.3, R Core Team, 2022) to analyze the collected data.

In each study, the prevalence of sleeping problems in Latin America was obtained. Studies that did not report the prevalence were excluded. The random effects model was used with the logit transformation for obtaining the pooled results, because it produces more conservative results than fixed-effects models, regardless of the heterogenicity scores [26]. The pooled prevalence estimates of sleeping problems in Latin America were presented as a percentage with 95% confidence intervals (CIs), using a forest plot. Also, a subgroup analysis was conducted for different sleep issues, cities, study quality, and age group to assess the contribution of each study to overall heterogeneity in the prevalence of sleep disturbance.

To measure the relationship between SES and sleeping problems, we extracted from the selected publications the adjusted odds ratio (aOR) with 95% confidence intervals (CIs). Their standard errors were calculated from the respective CIs. The value from each study and the corresponding standard error were transformed into their natural logarithms to stabilize the variances and to normalize the distribution. The pooled OR (and 95%CI) was estimated using a DerSimonian–Laird random effect model. In situations in which a study reported effect estimates for independent subgroups, the subgroups were treated as individual studies in the meta-analysis.

The test of the overall effect was assessed by using Z-statistics at *p* < 0.05. The heterogeneity among the included studies was assessed using Cochran’s Q test and I^2^ statistics. The thresholds 25%, 50%, and 75% were used to indicate low, moderate, and high heterogeneity, respectively [27,28]. A funnel plot based on Egger’s regression test was used to evaluate the publication bias [29]. In all analyses, a *p*-value less than 0.05 was considered statistically significant.

## 3. Results

### 3.1. Characteristics of Included Articles

Twenty articles were included [30,31,32,33,34,35,36,37,38,39,40,41,42,43,44,45,46,47,48,49] in the final sample, all of which are cross-sectional studies, and they are listed in Table 1. Among these studies, 80.0% (n = 16) were performed in Brazil [30,31,32,34,36,37,40,41,42,43,44,45,46,47,48,49], 10.0% (n = 2) were performed in Peru [38,39], 5.0% (n = 1) were performed in Chile [33], and 5.0% (n = 1) were multicentric (11 countries) [35]. The combined total number of participants was N = 128,455, comprising 3.7% (n = 4693) children, 16.0% (n = 20,586) adolescents [37,38,40,41,44], and 80.3% (n = 103,176) adults [30,31,32,33,34,35,36,38,39,45,46,47,48,49]. The smallest sample was n = 851 [42] and the largest was n = 60,202 [48]. The socioeconomic indicators used were income (monthly personal income, monthly family income, per capita income, and annual household income) [30,31,32,33,34,36,37,40,42,43,44,45,46], wealth/assets [36,38,39,48,49], number of residents in the household [45], employment status/occupation [30,31,33,34,36,37,38,45,46,49], accessed healthcare system [36], and composite indices [41]. The sleep variables used were excessive daytime sleepiness [31,39,48], sleep duration [36,37,38,40,43,44,45,49], sleep quality/sleep disturbance [32,33,35,37,43,46,48], insomnia [30,35], obstructive sleep apnea (OSA)/sleep-disordered breathing (SDB) symptoms [34,39], and bruxism [41,42,47]. A majority of the articles were of poor quality (55%), and a detailed qualitative evaluation is available in Table 2.

### 3.2. Descriptive Synthesis of Articles

Quantitative analyses are presented in the results section. Presented below is a descriptive analysis, which provides a deeper examination of the overall findings that were not considered in the quantitative analysis. Table 1 presents details of the individual studies included in the descriptive analysis.

#### 3.2.1. Sleep Duration

Seven cross-sectional studies examined the relation between socioeconomic status (SES) and sleep duration [36,37,38,40,43,45,49]. One of these was conducted with children (from 3 to 48 months old) [43], two studied adolescents (12 to 19 years old) [37,40], three involved adults [36,45,49], and one encompassed both adolescents and adults [38].

Overall, a higher SES was associated with a shorter sleep duration. Specifically, the highest level of education [36,37,38,40,45], being employed [36,37,38,45], higher income [37,38,40,45], and living with more residents [45] were associated with a shorter sleep duration in samples of adults and adolescents. In addition, long sleep was more prevalent among housewives [36], adolescents with black maternal skin color [40], and adolescents whose mothers had lower schooling [40].

In contrast, the poorest wealth index and being unemployed or not studying were associated with lower sleep percentage in another study with adults [49]. One study did not find consistent associations between sleep duration and maternal education or family income in children [43]. The overall quality of the selected studies was good for three studies [37,40,49] and fair for four studies [36,38,43,45].

#### 3.2.2. Sleep Quality/Sleep Disturbance

Eight cross-sectional studies assessed the relationship between sleep quality or disturbance and SES [32,33,35,37,43,44,46,48]. One of these studies was conducted with infants (from 3 to 48 months) [43], two with students (14 to 19 years old) [37,44], and the others with adults [32,46,48]. One study focused only on adult women [35]. The overall quality of the studies selected was good for three studies [35,37,44], fair for three studies [32,33,46], and poor for one study [48].

Globally, a lower SES was associated with diminished sleep quality. Specifically, low income [32,37] and unemployment [33,46] were associated with impaired sleep quality. Two studies indicated a higher prevalence of sleep disturbance in women [33,46]. More educated adults had significantly less sleep disturbances [32,35,48]. In contrast, one study found that higher maternal education was associated with lower quality of sleep in students [44], but this association was not found in infants in another study [43]. Additionally, psychiatric comorbidities [32,33] and alcohol and drug consumption [33,35] were also associated with sleep disturbance.

#### 3.2.3. Insomnia

Concerning insomnia, two cross-sectional studies assessed its relationship with SES [30,35]. One study evaluated only women [35], and one study assessed adults in general [30]. The overall quality of the two studies was good for one study [35] and fair for the other [30].

In both of the aforementioned studies, insomnia was independently associated with individuals with less education [30,35]. Moreover, alcohol and drug consumption was also associated with insomnia, according to another study [35].

#### 3.2.4. Excessive Daytime Sleepiness

Three cross-sectional studies approached the association between excessive daytime sleepiness and SES [31,39,48]. All studies assessed adults. The overall quality of the studies selected was good for one study [31], fair for another study [39] and poor for the third study [48].

Largely, excessive daytime sleepiness was associated with a lower SES in one the studies [39]. Additionally, it was also significantly more prevalent in individuals with a lower family income [31] and less education [48].

#### 3.2.5. Obstructive Sleep Apnea (OSA)/Sleep-Disordered Breathing (SDB) Symptoms

Concerning OSA/SDB, two cross-sectional studies assessed their relationship with SES [34,39]. Both studies evaluated adults in the general population. The overall quality was good in one study [34], and it was fair in the other study [39].

In one study, lower SES was associated with less frequent snoring. However, no significant association was found between SES and observed apneas [34]. The other study did not find any association between SES and OSA [39].

#### 3.2.6. Bruxism

Three cross-sectional studies assessed the relation between SES and bruxism [41,42,47]. One of the studies was conducted with infants (ages 6–12 years) [42], one with adolescents (aged 13–15 years) [41], and the last one with adults [47]. The overall quality was good in two studies [41,42] and fair in one study [47].

Among these three studies, two studies reported that sleep bruxism was independently associated with higher SES, including higher education [41,47]. However, the other study, which was conducted by Mota-Veloso et al. [42], found that SES had a significant indirect effect on bruxism via sucking habits (finger sucking and biting nails or other objects) and that a lower SES was associated with more sleep bruxism [42].

### 3.3. Prevalence of Sleep Disturbances in Latin America

**Figure 2:**
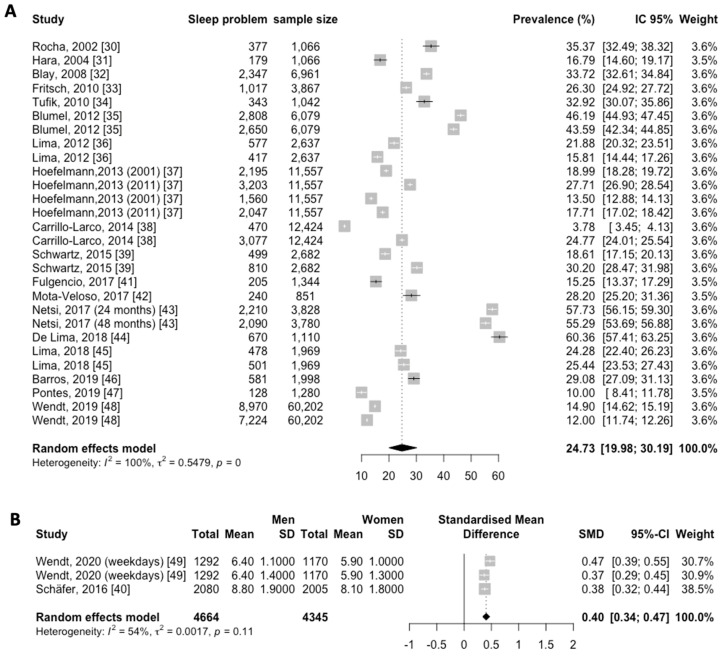
Forest plot showing the primary outcomes in 31 cross sectional studies from 20 published reports in Latin America: (**A**) prevalence of sleep disturbances and (**B**) sleep length (hours).

Based on our selection criteria, eighteen studies [30,31,32,33,34,35,36,37,38,39,41,42,43,44,45,46,47,48] were eligible. Whenever results in the same article were reported for different sleep disturbance types separately, they were entered into the analysis as separate studies. Therefore, a total of 18 papers including 28 studies were included in the final meta-analysis, which is shown in Figure 2A.

**Figure 3:**
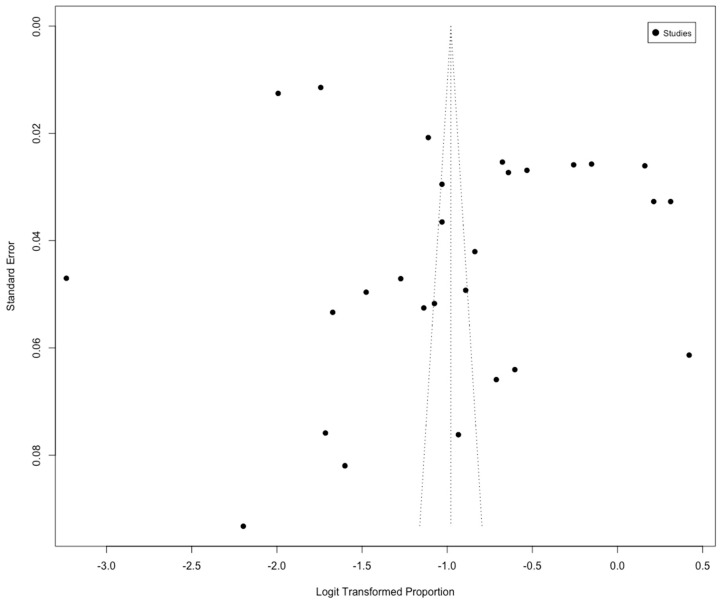
Funnel plot for meta-analysis of the prevalence of sleep disturbances in Latin America. Egger’s test: *p* = 0.0598.

The overall pooled prevalence for sleep disturbance in Latin America was 24.73% (95% CI, 19.98–30.19; I^2^ = 100%) (Figure 2A). To decide whether to include all of the articles examining sleep disturbances or not, a publication bias chart was created. The results showed that publication bias was not significant (*p* = 0.059) (Figure 3).

**Figure 4:**
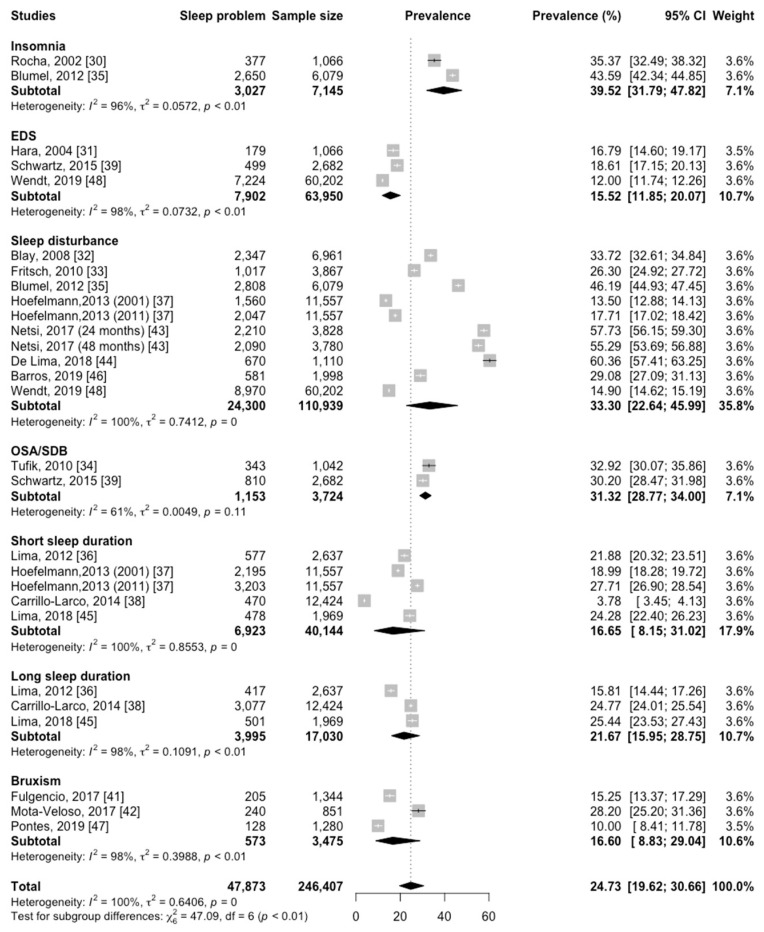
Subgroup analysis on the prevalence of sleep disturbances in Latin America by type of sleep disturbance. The black dot point is the estimate, and the horizontal line is the 95% CI for prevalence plotted for each study. The black diamond at the bottom of each type of sleep disturbance is the estimated average prevalence. CI: confidence interval.

We divided eighteen papers into seven categories according to different types of sleep disturbances. The subgroup analysis was manipulated based on seven categories (Figure 4). The highest prevalence was for insomnia, with 39.52% (95% CI, 31.79–47.82; *p* < 0.01), and the lowest prevalence was for EDS, with 15.52% (95% CI, 11.85–20.07; *p* < 0.01). The overall pooled prevalence for sleep disturbance was 33.30% (95% CI, 22.64–45.99; *p* = 0) across eight articles [32,33,35,37,43,44,46,48]; for OSA/SDB, it was 31.32% (95% CI, 28.77–34.00; *p* = 0.11) across two articles [34,39]; and for bruxism, it was 16.60% (95% CI, 8.83–29.04; *p* < 0.01) across three articles [41,42,47]. The overall pooled prevalence for short sleep duration was 16.65% (95% CI, 8.15–31.02; *p* = 0) across four articles [36,37,38,45], and for long sleep duration, it was 21.67% (95% CI, 15.95–28.75; *p* < 0.01) across three articles [36,38,45].

### 3.4. Sleep Length in Latin America

A total of two articles [40,49] reported the sleep length (Figure 2B). Where results were reported for men and women separately, they were entered into the analysis as separate studies. In the pooled analysis, sleep length was higher in men with sleep disturbances than women without any significant differences, with a standardized mean of 0.40 h (95% CI, 0.34–0.47; *p* = 0.11; I^2^ = 100%).

### 3.5. Subgroup Analysis

Because of a high level of heterogeneity across the included studies, a subgroup analysis was performed by region (cities), age group, and quality of study in relation to the principal outcome variable. The analysis revealed that the prevalence of sleep disturbances among infants, i.e., 56.52 (95% CI 54.11–58.89), was greater than that of adolescents, i.e., 20.33 (95% CI 12.68–30.95), and that of adults, i.e., 24.24 (95% CI 18.93–30.48). Also, the pooled prevalence of sleep disturbances was higher in Brazil, 25.00 (95% CI 19.54–31.40), than in Peru, 15.91 (95% CI 6.17–35.27) (Table 3).

### 3.6. Risk Factors

A meta-analysis was possible for sleep disturbance prevalence with three SES factors (education, income, and employment status).

#### 3.6.1. Education and Sleep Disturbances

**Figure 5:**
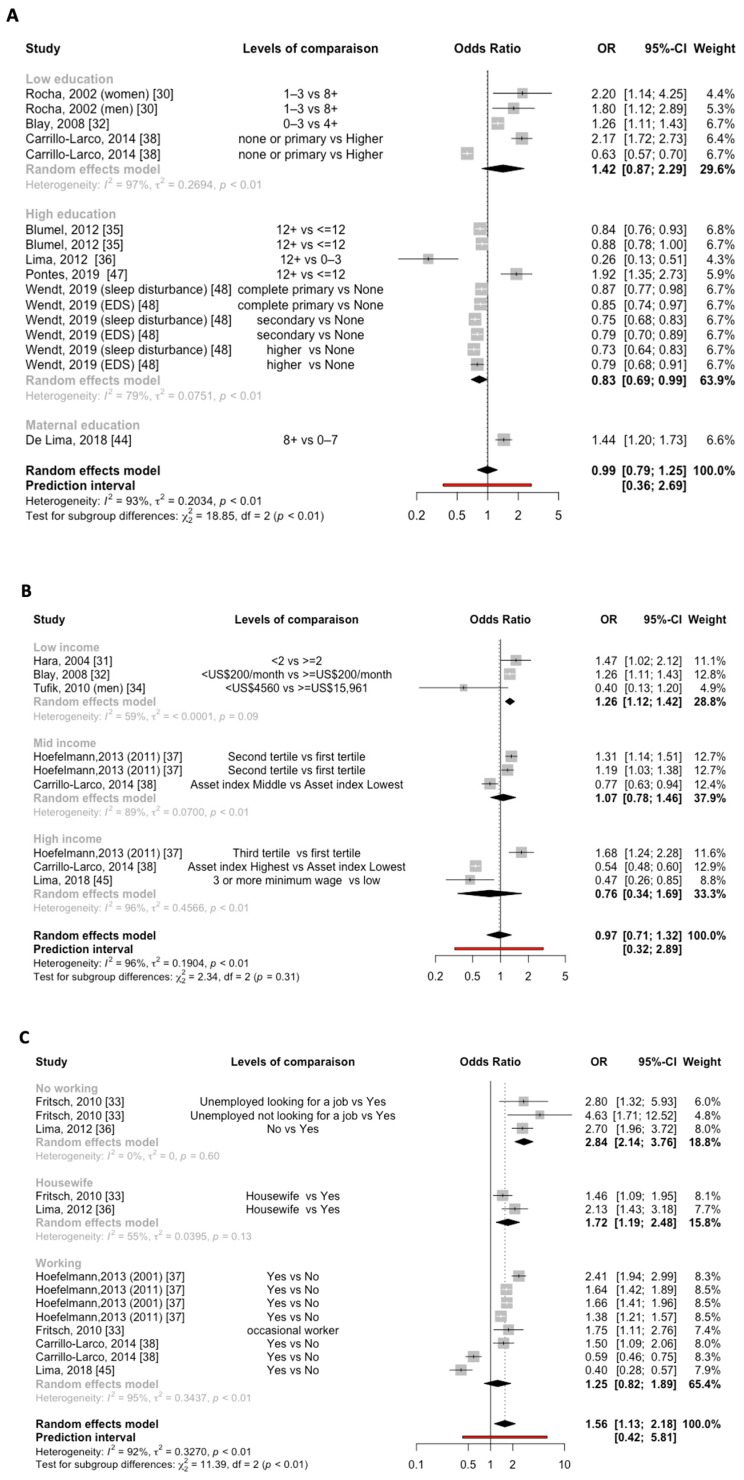
(**A**) Forest plot for education and sleep disturbance prevalence (compared to the reference group). (**B**) Forest plot for income and sleep disturbance prevalence (compared to the reference group). (**C**) Forest plot for employment status and sleep disturbance prevalence (compared to the reference group). Box sizes reflect the weights of studies included in the meta-analysis, horizontal lines are the 95% CIs, and the summary ORs are represented by the diamond. OR, odds ratio; CI, confidence interval.

Eight studies [30,32,35,36,38,44,47,48] evaluated the association between education and the prevalence of sleep disturbances, as shown in Figure 5A. No association was found between low education and prevalent sleep disturbances, with a pooled OR of 1.42 (95%CI, [0.87–2.29]; *p* < 0.01). However, the meta-analysis showed that the highly educated population had a lower prevalence of sleep disturbances (OR, 0.83; 95%CI, [0.69–0.99]; *p* < 0.01), with a high heterogeneity between studies (I^2^ = 79%).

**Figure 6:**
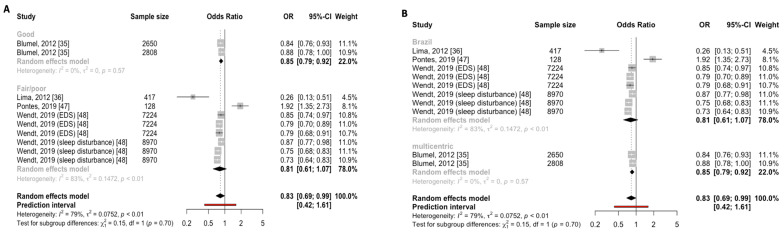
Subgroup analyses to explore sources of heterogeneity in risk factors of sleep disturbances. (**A**) Forest plot demonstrating that higher education was associated with sleep disturbance prevalence by quality of the study (good vs. fair/poor)**.** (**B**) Forest plot demonstrating that higher education was associated with sleep disturbance prevalence by city (Brazil vs. multicentric).

Separating the education analyses according to the quality of the studies did not reveal a significant subgroup effect for sleep disturbance prevalence (*p* = 0.70; Figure 6A). Similarly, when the education analyses were separated according to city, no significant subgroup effect for the prevalence of sleep disturbances was observed (*p* = 0.70; Figure 6B).

#### 3.6.2. Income and Sleep Disturbances

Six studies [31,32,34,37,38,45] considered income as a risk factor of sleep disturbances in Latin America. The meta-analysis showed a significant relationship between low income and the prevalence of sleep problems (OR, 1.26; 95%CI, [1.12–1.42]), with moderate heterogeneity between studies (I^2^ = 59%). However, it did not show a significant association between mid-income (OR, 1.07; 95%CI, [0.78–1.46]; *p* < 0.01) or high income (OR, 0.76; 95%CI, [0.34–1.69]; *p* < 0.01) and prevalence of sleep problems (Figure 5B).

#### 3.6.3. Work and Sleep Disturbances

Data on the relationship between work and sleep disturbances are shown in Figure 5C. The meta-analysis showed a significant relationship between unemployment and the prevalence of sleep disturbances (OR, 2.84; 95%CI, [2.14–3.76]), with no heterogeneity between studies (I^2^ = 0%). Similarly, in the pooled analysis, being a housewife was associated with a high prevalence of sleep disturbances (OR, 1.72; 95%CI, [1.19–2.48]), with moderate heterogeneity between studies (I^2^ = 55%).

## 4. Discussion

### 4.1. Detailed Summary of Findings

The sleep outcomes analyzed were sleep duration, sleep quality/sleep disturbance, insomnia, excessive daytime sleepiness (EDS), obstructive sleep apnea (OSA)/sleep-disordered breathing (SDB) symptoms, and bruxism. The most used determinants were income, education level, employment status/occupation, wealth/assets, and composite indices.

A higher SES was associated with lower sleep duration, and a lower SES was associated with a decrease in sleep quality, less frequent snoring, more prevalent EDS, and sleep bruxism. Lower education was associated with insomnia, and higher education was associated with more sleep bruxism. For the 20 articles included, 12 were rated as fair or poor in study quality. Therefore, a meta-analysis was performed to estimate the prevalence of sleep disturbances in Latin America and the main SES risk factors that could be associated with it. The pooled prevalence, using a meta-analysis of the random effects model, was 24.73% (95%CI, 19.98–30.19), with high heterogeneity (I^2^ = 100%). The meta-analysis showed that the prevalence of sleep disturbances decreased with high education (OR, 0.83; 95%CI, [0.69–0.99]; I^2^ = 79%), while it increased with low income (OR, 1.26; 95%CI, [1.12–1.42]; I^2^ = 59%), unemployment (OR, 2.84; 95%CI, [2.14–3.76]; I^2^ = 0%), and being a housewife (OR, 1.72; 95%CI, [1.19–2.48]; I^2^ = 55%).

### 4.2. Relationship with Public Health Literature

Epidemiologic data continue to increase the literature about the influence of sleep on the general population’s health status. Sleep plays a vital role in several body functions, as well as health disparities. The scientific community is still investigating external and environmental factors affecting sleep mechanisms, but there is still a lot that is unknown. Based on the current findings, it can be hypothesized that sleep disturbances are associated with socioeconomic status, as suggested by many other studies [17,50,51,52,53,54,55,56,57]. The fact is that the gradient of health disparity existing for some diseases, like cardiovascular illness, seems the same for sleep. Regardless of the region of the world where the investigation is conduced, sleep disparities are observed because our findings on Latin America support the previous results [17,50,51,52,53,54,55,56,57]. Our findings are consistent with those of previous studies from places other than Latin America, and they are additional arguments in favor of the establishment of a more efficient worldwide program framing sleep health management.

### 4.3. The Necessity of a Multidimensional Sleep Management

**Figure 7:**
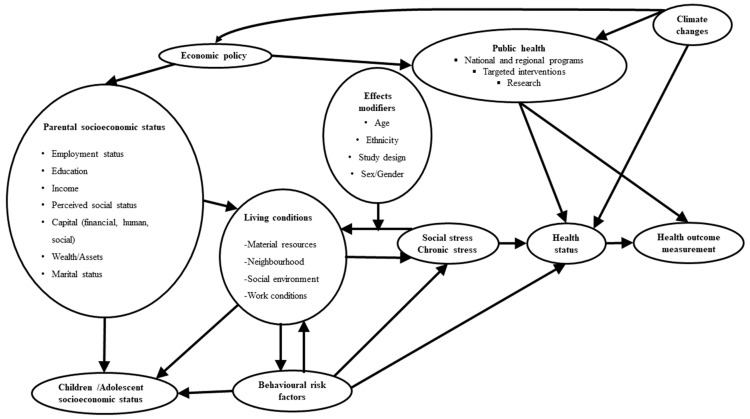
The Socioeconomic and Environmental Model of Health (SEEMOH).

Sleep disturbance management should be addressed by a multidimensional approach. Recent epidemiological studies performed outside Latin America in different public health contexts reported significant associations of sleep with stress [4], work conditions [8], environment [58], and employment [17,59,60], and they also revealed latent interactions existing between government policy and public health strategies [22,54,61,62,63] (Figure 7). Obviously, a government’s economic policy influences the funding of public health programs. Similarly, SES directly influences health status regardless of the disease, as assessed through individuals’ living conditions and their resulting behavioral risk factors and stress. Knowing that sleep disparities can be measured objectively and quantitatively [6,7,49], our suggestion for governments is to invest as soon as possible in preventive management programs for sleep disturbances before they become uncontrollable. It was already documented how expensive absenteeism and presenteeism due to sleep disturbances are for the economy [64,65], but diverse governments did not move forward yet with strong regulations to reduce these important losses [66,67].

In this meta-analysis, there is an inequal distribution of research because 80% of studies came from Brazil (Table 1). Even if Brazil is representative of Latin American populations, its public health’s context regarding sleep management is not necessarily identical to that of its neighbors. More research should be performed in other Latin American countries to obtain an accurate overview of sleep disparities in this continent. Our suggestion for scientists is to not forget that cross-sectionals studies are often used to understand determinants of health and establish preliminary evidence [68]; however, they are useless when it is necessary to consider the correlation between theoretical determinants and health outcomes. This first meta-analysis on sleep determinants in Latin America highlighted the high quality of cross-sectional studies published, as well as the lack of systematic review and longitudinal studies, similar to what has been found recently with the African population [69]. To support public health strategies, randomized controlled trials and longitudinal studies are required with a broader objective related to the SES–sleep health gradient, including the role of unhealthy behaviors, chronic diseases, and psychological factors [68].

## 5. Conclusions

This meta-analysis reveals that the prevalence of sleep disturbances accounts for almost a quarter of the health issues in Latin America, and they have been associated with lower SES, especially in terms of education, income, and work. Despite the growing body of literature around the importance of sleep, it seems non-significant enough for decision makers who still do not pay attention to this public health matter. Governmental programs should consider scientific evidence and could be funded to allow fast and permanent results in the near future, before reaching an uncontrollable point.

## Figures and Tables

**Table 1:** Characteristics of included studies investigating determinants of sleep health in Latin America.

Study	Study Design	Population	% Women	Age (Years)	Sample Size	SES Measures	Sleep Measures	Statistically Significant Findings	Main Effects	Interactions/Mediations
Rocha 2002 [30]	Cross-sectional	Adults from Bambui, Brazil	55.5	39 (N/R)	1066	Years of education (0, 1–3, 4–7, 8+) Current employment situation (working, not working, retired) Monthly family income (<2.0, 2.0–3.9, ≥4 Brazilian minimum wage)	Insomnia symptoms (difficulty initiating sleep, frequency of disrupted sleep, and frequency of early morning awakening) during the last 30 days, at least three times a week or more, with any level of distress	Insomnia was higher in females with age 60+ (OR, 1.8; CI, 1.1–1.3) and with 1–3 or no years of schooling (OR = 1.8, CI = 1.1–3.0; OR = 2.6, CI = 1.3–5.1) and males with 1–3 years of schooling (OR, 2.2; CI, 1.1–4.1)	Insomnia was independently associated with less education	
Hara 2004 [31]	Cross-sectional	Adults from Bambui, Brazil	55.5	39 (N/R)	1066	Years of education (0, 1–3, 4–7, 8+) Monthly personal income (none, <1, 1.0–1.9, ≥2.0 Brazilian minimum wages) Monthly family income (<2.0, ≥2.0 Brazilian minimum wages) Current employment situation (student, working, unemployed, retired)	Excessive daytime sleepiness three or more times per week with consequent impairment of daily activities	EDS was associated with insomnia (OR, 2.24; CI, 1.6–3.15) and lower family income (OR, 1.47; CI, 1.02–2.12)	Lower family income was associated with EDS	
Blay 2008 [32]	Cross-sectional	Adults ≥ 60 y from Rio Grande do Sul, Brazil	66.0	N/R	6961	Income (< vs. ≥USD 200/month) Years of education (0–3 vs. ≥4)	Sleep disturbance (single question, yes vs. no) in the last 30 days	Lower income (OR, 1.26; CI, 1.11–1.43) and lower education (OR, 1.8; CI, 1.24–1.76) were related to disturbed sleep	Low income and low education were independent risk factors for self-reported sleep disturbance.	
Fritsch Montero 2010 [33]	Cross-sectional	Adults 18–64 y from Gran Santiago, Chile	52.3	N/R	3867	Schooling (upper, middle, basic, none) Employment status (10 levels) Per capita income quartiles (high, middle high, middle low, low), decrease of income.	Revised Clinical Interview Schedule sleep score > 1	Being an occasional worker (OR, 1.75; CI, 1.11–2.77), unemployed and looking (OR, 2.8; CI, 1.32–5.93) and not looking (OR, 4.63; CI, 1.7–12.51) for a job, having adequate (OR, 2.05; CI, 1.21–3.47) and poor living conditions (OR, 2.04; CI, 1.24–3.47), being a housewife (OR, 1.46; CI, 1.09–1.95) or female (OR, 1.43; CI, 1.14–1.80) were considered to be risk factors for sleep disturbance	Unemployed and occasional workers patients, housewives, patients with common mental disorders had higher odds of having sleep disorders	
Tufik 2010 [34]	Cross-sectional	Adults 20–80 y from the general population in Sao Paulo, Brazil	55	N/R	1042	Annual household income (high, mid, low according to the Brazilian Economic Classification Criteria), Employment status (working vs. not working)	OSA ICSD-2 criteria (AHI from PSG, items 2 and 5 from Berlin Questionnaire, ESS > 10 and/or item 8 from PSQI, Chalder Fatigue Scale > 4)	Increasing age (OR, 3.9; 6.6; 10.8; 34.5) and gender (OR, 4.1; CI, 2.9–5.8) were independent and strong associated factors for the presence of OSA.	SES was not associated with OSA	Low income was a protective factor for males (OR, 0.4; CI, 0.1–0.9), but not significant in females (*p* = 0.057)
Blümel 2012 [35]	Cross-sectional	40–59 y women recruited from 20 healthcare centers in 11 Latin American countries	100	49.8 (5.4)	6079	Education (≤ vs. >12 years),	Insomnia (AIS score > 5). Sleep quality (PSQI global score > 5).	Education > 12 years was associated with less insomnia (OR, 0.84; CI, 0.74–0.9) and less poor sleep quality (OR, 0.83; CI, 0.73–0.94).	Higher educational level was an independent risk factor related to less insomnia and better sleep quality	
Lima 2012 [36]	Cross-sectional	Adults from general population in Campinas, Brazil	52.3	41.8 (95% CI: 40.7–42.9)	2637	Education (0–3, 4–7, 8–11, ≥12 y), per capita monthly household income (1 minimum salary or less, 1–3 times the minimal salary, 3 or more times the minimum salary), work status (working, not working, housewife), number of household appliances (≤10, >10).	Sleep duration (≤6, 7–8, ≥9 h)	Long sleep (≥9 h) was less prevalent among those with 4–11 or ≥12 years of schooling (OR = 0.38, CI = 0.25–0.60; OR = 0.26, CI = 0.13–0.50) and more prevalent among those not working or housewives (OR = 2.70, CI = 1.96–3.73; OR = 2.13, CI = 1.43–3.18)	Long sleep was more prevalent among those with a lower level of education, those who did not work and housewives.	
Hoefelmann 2013 [37]	Cross-sectional	Adolescents 15–19 years from high-schools in Santa Catarina, Brazil	57.2	15–19 years	5932 (2001) and 5932 (2011)	Work status (yes vs. no), monthly family income (in terciles), school grading and school shift	Insufficient sleep duration (<8 h), poor sleep quality (sometimes/hardly ever/never vs. always/nearly always)	Working (OR = 1.66, CI = 1.41–1.96; OR = 1.38, CI = 1.21–1.57 in 2001 and 2011, respectively), school grade (OR third year in 2001 = 1.42; CI, 1.02–1.99), income (OR second tercile in 2011 = 1.19; CI, 1.03–1.38), and school shift (OR night in 2011 = 1.26; CI, 1.07–1.49) were related to poor sleep quality. Working (OR = 2.41, CI = 1.94–2.99; and OR = 1.64, CI = 1.42–1.89 in 2001 and 2011, respectively), monthly family income (OR third tercile in 2011 = 1.68; CI, 1.24–2.28) and higher school grading (OR third year in 2011 = 1.48; CI, 1.15–1.91) were associated with insufficient sleep duration.	Working and higher family income was associated with both short sleep and poor sleep quality. Higher school grading was associated with both short sleep and poor sleep quality. Night school shift was associated with poor sleep quality.	
Carrillo- Larco 2014 [38]	Cross-sectional	Adolescents and adults ≥ 12 y from the general population of Peru	49.4	35.8 ± 17.7	12,424	Education (none/primary school, high school, higher), asset index (in tertiles), job status (yes vs. no)	Self-reported sleep duration (short-sleep < 6, regular-sleep 6–8, long-sleep > 8 h)	Higher probability of being a short-sleeper was found in those currently employed (OR, 1.5; CI, 1.09–2.06) and lower probability in those with high school (OR, 0.53; CI, 0.32–0.86) or none/primary education (OR, 0,63; CI, 0.57–0.7). Higher probability of being a long-sleeper was found in those with high school (OR, 1.42; CI, 1.34–1.51) or none/primary education (OR, 2.17; CI, 1.72–2.73) and lesser probability in those with middle (OR, 0.77; CI, 0.63–0.94) or highest (OR, 0.54; CI, 0.48–0.60) assets index and currently working (OR, 0.59; CI, 0.46–0.75).	Participants with lower education were more likely to have long sleep duration and less likely to have short sleep duration. Those with higher asset index were less likely to report long sleep. Employed individuals had a higher probability of being short sleepers and lower probability of being long sleepers than unemployed.	
Schwartz 2015 [39]	Cross-sectional	Adults > 35 y from the general population of 4 Peruvian settings	49.5	54.1 (18.8)	2682	Wealth index—based on current occupation, household income, assets, and household facilities (in tertiles)	SDB symptoms: habitual snoring (self-reported snoring at least 3 nights per week); observed apnea (pauses in breathing or choking during sleep reported by a spouse or bed partner); excessive daytime sleepiness (modified ESS score > 6)	More excessive daytime sleepiness was associated with medium SES (OR, 1.41; CI, 1.10–1.80; *p* = 0.006). Less habitual snoring was associated with medium (OR, 0.79; CI, 0.64–0.97; *p* = 0.027) and low (OR, 0.7; CI, 0.55–0.90; *p* = 0.005) SES.	Lower SES was associated with less habitual snoring but more excessive daytime sleepiness. No significant association was found between SES and observed apnea.	
Schäfer 2016 [40]	Cross-sectional	Adolescents 18 y members of a population-based birth cohort in Pelotas, Brazil	50.9	18 (N/R)	4016	Family income at birth and at 18 y (in quintiles) Maternal schooling in completed years at birth (0, 1–4, 5–8, 9–11, ≥12 y) Maternal skin color (white, black, other) Currently enrolled in school (yes, no) Adolescent schooling in completed years (≤4, 5–8, ≥9 y)	Self-reported sleep duration (h/day)	Maternal schooling was associated with sleep duration in both genders, with an inverse linear trend in girls (*p* < 0.001). Girls whose mothers had no schooling showed an increase of 1.40 (ß) hours per day (95%CI, 0.77–2.04) compared to those whose mothers had ≥12 y of schooling. Girls whose mothers were black had 0.37 h more sleep per day (95%CI, 0.17–0.58) than those whose mothers were white. Girls in the lowest fifth of family income at birth had a higher sleep duration (ß = 0.58; 95%CI, 0.30–0.87) compared to girls in the highest fifth. Adolescents who were currently studying showed lower sleep duration (ß = −0.34; 95%CI, −0.51 to −10.17 for males; ß = −0.75, 95%CI, −0.92 to −0.59 for females). Boys with lower schooling showed higher sleep duration (ß = 0.45; 95%CI, 0.09–0.81 for males). Girls with intermediate schooling (5–8 years) showed higher sleep duration (ß = 0.46; 95%CI, 0.27–0.65). Boys and girls in the lowest fifth family income at 18 years had higher sleep duration (ß = 0.58; 95%CI, 0.33–0.84) and (ß = 0.69; 95%CI, 0.41–0.96), respectively	Lower maternal and adolescent schooling and lower family income was associated with higher sleep duration. Black maternal skin color was associated with higher sleep duration in girls.	
Fulgencio 2017 [41]	Cross-sectional	Adolescents 13–15 y from 14 public and private schools in Itabira, Brazil	56.1	13–15 years	1344	SES—composite variable (goods owned by the family and educational level of its head), categorized as higher vs. lower	Parent-reported possible sleep bruxism (single question, yes vs. no)	Higher SES was associated with higher prevalence of possible sleep bruxism (PR, 1.51; 95%CI, 1.23–1.86)	Greater prevalence of possible sleep bruxism was observed among adolescents with a higher SES	
Mota-Veloso 2017 [42]	Cross-sectional	Children 6–12 y from seven public and two private schools in Diamantina, Brazil	54.8	6–12 years	851	SES—composite variable of 3 indicators: equivalized household income (10 levels), mother’s and father’s schooling (9 levels)	Sleep bruxism (reports of parents/caregivers and oral clinical evaluation)	SES had a significant indirect effect on bruxism via sucking habits (SC = −0.08; *p* = 0.01). SES had a significant direct effect (SC = −0.16; *p* = 0.01) and the total effect on tooth wear was also significant (SC = −0.17; *p* = 0.00).	Lower SES was associated with more sleep bruxism	The effect was mediated by sucking behavior (finger sucking, biting nails or other objects)
Netsi 2017 [43]	Cross-sectional	Infants from Pelotas, Brazil	N/R	N/R	3842	Maternal education (0–4 years, 5–8 years, ≥9 years) Family income (in quintiles)	Parent-reported sleep duration, awakenings, and sleep disturbances (nightmares/night terrors, restless sleep, difficulty going to sleep, wakes up at night, and wakes up early)	There were no consistent associations between sleep duration or sleep disturbances and sociodemographic characteristics	Maternal education and family income were not associated with infant sleep duration or disturbances	
De Lima 2018 [44]	Cross-sectional	Students with 14–19 y, from high schools in São José, Brazil	54.2	16.1 ± 1.1	1110	Maternal education (<8 vs. ≥8 years) Family income (up to two minimum wages; two to ten times the minimum wage; more than ten times the minimum wage)	Sleep quality (perception of sleep quality, single question, categorized in almost never/seldom/sometimes vs. with relative frequency/almost always)	The prevalence of low quality of sleep was higher in adolescents whose mothers had up to 8 years of study (OR, 1.44; CI, 1.13–1.84). Those who had sedentary behavior of risk based on screen time (OR, 0.54; CI, 0.42–0.70).	Students whose mothers had a high level of education were more likely to have a low quality of sleep. Students with sedentary risk behavior were less likely to report poor sleep quality.	
Lima 2018 [45]	Cross-sectional	Adults ≥ 20 y from the general population of Campinas, Brazil	52.7	43.7 (CI 42.3–45.2)	1969	Work status (yes vs. no) Per capita family income (<1; 1–2; 3 or more minimum wages) Schooling (0–4; 5–8; 9–11; 12 or more years of schooling) Number of residents in the household (1;2;3 or more)	Sleep duration (6 h or less; 7–8 h; 9 h or more)	Associated with short sleep: highest level of schooling (OR, 1.73; CI, 1.08–2.75). The probability of long sleep was lower in individuals who work (OR, 0.39; CI, 0.28–0.55), with higher income (OR, 0.49; CI, 0.29–0.85)	Those with higher schooling were more likely to have short sleep. The chance of long sleep was lower in those who have more years of schooling, have higher income, and worked.	Adjusting for chronic diseases and health disturbances attenuated the effects of education on short sleep
Barros 2019 [46]	Cross-sectional	Adults ≥ 20 y from the general population in Campinas, Brazil	52.7	43.7 (CI 42.3–45.3)	1998	Education (0–3 y, 4–8 y, 9–11 y, ≥12 y) Per capita family income (<1 minimum wage, 1–3, >3) Employment (working vs. not working)	Sleep quality (single question; excellent/ very good/good vs. regular/poor/very poor)	Poor sleep quality was more frequent in women (OR, 1.36; CI, 1.14–1.63), older individuals (OR, 1.5; CI, 1.20–1.87), unemployed (OR, 1.26; CI, 1.03–1.54), and in those with the highest number of children (OR, 1.33; CI, 1.02–1.74).		Adjusting for amount of health disturbances, self-rated health, common mental disorders, and life satisfaction attenuated the effect
Pontes 2019 [47]	Cross-sectional	Adults ≥ 18 y from the general population in Rio Grande, Brazil	56.6	45.9 ± 17.2	1280	Education (0 to 11 years vs. 12 years or more)	Sleep bruxism (ICSD criteria)	In the bivariate analysis, the schooling (OR, 1.66; CI, 1.14–2.42) and stress (OR, 1.66; CI, 1.14–2.42) variables were associated with sleep bruxism. In the adjusted analysis, age (OR, 1.63; CI, 1.07–2.46), schooling (OR, 1.92; CI, 1.35–2.72), and stress (OR, 1.76; CI, 1.11–2.81) were associated with sleep bruxism.	Higher education and psychological stress were associated with higher prevalence of sleep bruxism	
Wendt 2019 [48]	Cross-sectional	Adults from the general population in Brazil	52.9	N/R	60,202	Education (none, incomplete primary level, complete primary level, secondary level, higher education) Wealth index (assets index score, in quintiles)	Sleep disturbance frequency and daytime fatigue in last two weeks (none; up to seven days; more than seven days; almost every day)	Sleep disturbances and daytime fatigue had lower prevalence in highly educated individuals with, respectively, (OR, 0.73; CI, 0.64–0.83) and (OR, 0.79; CI, 0.69–0.92)	Highly educated individuals had lower prevalence of sleep disturbance than those with no formal education	
Wendt 2020 [49]	Cross-sectional	22-year-old adults from a population-based birth cohort in Pelotas, Brazil	53.2	22 (N/R)	2462	Wealth index (asset index, in quintiles), occupation (none, only study, only work, both)	Sleep duration and efficiency (7-day accelerometry)	Women not working or studying presented higher Sleep Time Window (OR, 7.5; CI, 7.3–7.6) and lower Sleep Percent (OR, 82; CI, 81.1–83.7). Those in the poorest quintile of wealth index presented lower SP (OR, 82.4; CI, 81.9–83.7)	Women in the poorest quintile of wealth index presented with lower sleep efficiency	

AHI = apnea-hypopnea index; AIS = Athens Insomnia Scale; CI = confidence interval; EDS = excessive daytime sleepiness; ESS = Epworth Sleepiness Scale; ICSD = International Classification of Sleep Disorders; OR = odds ratio; OSA = obstructive sleep apnea; PSG = polysomnography; PSQI = Pittsburgh Sleep Quality Index; SDB = sleep-disordered breathing; SES = socioeconomic status. Age is presented as mean ± standard deviation or median (interquartile range or range).

**Table 2:** Quality rating of the included studies, using the NIH quality assessment tool.

Study	Q1	Q2	Q3	Q4	Q5	Q6	Q7	Q8	Q9	Q10	Q11	Q12	Q13	Q14	Quality Rating
Rocha 2002 [30]	Y	Y	Y	Y	Y	N	N	Y	N	N	Y	NA	NA	Y	Fair
Hara 2004 [31]	Y	Y	Y	Y	Y	N	N	Y	N	N	Y	NA	NA	Y	Fair
Blay 2008 [32]	Y	Y	Y	Y	Y	N	N	Y	Y	N	N	NA	NA	Y	Fair
Fritsch Montero 2010 [33]	Y	Y	N	Y	N	N	N	Y	Y	N	Y	NA	NA	Y	Fair
Tufik 2010 [34]	Y	Y	Y	Y	Y	N	N	Y	Y	N	Y	NA	NA	Y	Good
Blümel 2012 [35]	Y	Y	Y	Y	Y	N	N	Y	Y	N	Y	NA	NA	Y	Good
Lima 2012 [36]	Y	Y	Y	Y	Y	N	N	Y	N	N	N	NA	NA	Y	Fair
Hoefelmann 2013 [37]	Y	Y	Y	Y	Y	N	N	Y	N	Y	Y	NA	NR	Y	Good
Carrillo-Larco 2014 [38]	Y	Y	Y	Y	N	N	N	Y	Y	N	Y	NA	NA	Y	Fair
Schwartz 2015 [39]	Y	Y	Y	Y	N	N	N	Y	Y	N	Y	NA	NA	Y	Fair
Schäfer 2016 [40]	Y	Y	Y	Y	N	N	N	Y	Y	Y	N	N	Y	Y	Good
Fulgencio 2017 [41]	Y	Y	Y	Y	Y	N	N	Y	Y	N	Y	NA	NA	Y	Good
Mota-Veloso 2017 [42]	Y	Y	Y	Y	Y	N	N	Y	Y	N	Y	NA	NA	Y	Good
Netsi 2017 [43]	Y	Y	Y	Y	N	N	N	Y	N	Y	N	N	Y	Y	Fair
De Lima 2018 [44]	Y	Y	Y	Y	Y	N	N	Y	Y	N	Y	NA	NA	Y	Good
Lima 2018 [45]	Y	Y	Y	Y	Y	N	N	Y	Y	N	N	NA	NA	Y	Fair
Barros 2019 [46]	Y	Y	Y	Y	Y	N	N	Y	Y	N	N	NA	NA	Y	Fair
Pontes 2019 [47]	Y	Y	Y	Y	Y	N	N	N	Y	N	Y	NA	NA	Y	Fair
Wendt 2019 [48]	Y	Y	Y	Y	Y	N	N	N	N	N	N	NA	NA	Y	Poor
Wendt 2020 [49]	Y	Y	Y	Y	Y	N	N	Y	Y	N	Y	NA	N	Y	Good

N = no; NA = Not applicable; NR = Not Reported; Y = Yes.

**Table 3:** Subgroup analyses of the prevalence of sleep disturbances in Latin America.

Subgroup	Number of Studies	Pooled Prevalence (95 CI%)	I^2^ (%)
Total	28	24.73 [19.62–30.66]	100
Cities			
Brazil	21	25.00 [19.54–31.40]	100
Chile	1	-	-
Peru	4	15.91 [6.17–35.27]	100
Multicentric	2	44.89 [42.36–47.45]	88
Age group			
Adult	15	24.24 [18.93–30.48]	100
Adolescents	10	20.33 [12.68–30.95]	100
Children	1	-	-
Infant	2	56.52 [54.11–58.89]	78
Study’s quality			
Good	12	27.97 [20.59–36.76]	100
Fair	14	24.08 [16.61–33.55]	100
Poor	2	13.38 [10.79–16.49]	100

## Data Availability

Not applicable.
